# Now You Don't See It, Now You Do: Filling In Creates the Illusion of Motion

**DOI:** 10.1371/journal.pbio.0030290

**Published:** 2005-07-19

The parade of lights flashing on a theater marquee provides an important lesson in how the brain creates the illusion of motion. While we know each bulb remains stationary, the lighting and dimming of each in succession makes it appear that light is moving across the marquee. Even when successive bulbs are separated by a large space, our brains fill in the missing data to create the illusion that the motion has occurred smoothly from one point to the next. But where in the brain does the illusion occur? In this issue of *PLoS Biology*, Lars Muckli and colleagues demonstrate that the first cortical area in the visual processing stream, the primary visual cortex, participates in sustaining the illusion, probably under the influence of higher visual centers in the brain that likely create it.

In this study, human subjects observed a simple white square on a computer screen, flashed first in one spot, and then again several centimeters away. A functional magnetic resonance image of their brain activity was recorded, and the most active areas were determined. In the primary visual cortex, or V1, activity was found in one area that corresponded to the location in the visual field of the first flash, and in another area corresponding to the location of the second flash. Remarkably, there was also activity in between these two locations, corresponding to a region of the visual field that, while not itself illuminated, was on the path between the two flashes. This same area was active when the subjects viewed the white square moving smoothly between the two spots, and was absent when the square was flashed at only the initial or only the final spot, or when squares in both spots flickered simultaneously. The subjects' brains, it seems, filled in the missing information when a pattern of activity suggesting motion was detected. Further evidence that such “fill-in” activity in V1 corresponds to the conscious perception of motion came from an additional experiment, in which the flashing pattern of a quartet of white squares suggested alternating, and mutually exclusive, horizontal or vertical motion. When the observers reported the quartet as moving vertically, there was more activity in the V1 region corresponding to the vertical apparent motion.

The source of this filling in is not likely to be in V1 itself, the authors argue, because the middle area is too far from either of the ends to be stimulated by them directly. Instead, they propose the most likely source is activity in a visual area much higher in the processing chain, called hMT/V5+. Projections from here are known to influence V1 activity, and cover large enough areas to encompass the entire region of V1 activation in these experiments. [Fig pbio-0030290-g001]

**Figure pbio-0030290-g001:**
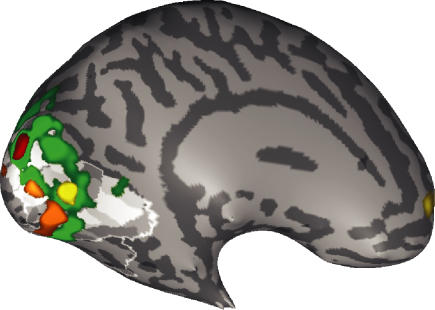
The brain regions highlighted above allow us to see a series of flashing images as smooth motion. This activity occurs in the primary visual cortex

These results provide further demonstration that the brain masterfully creates a continuous, yet sometimes imagined, whole from individual, and often incomplete, parts. Most surprisingly, even the brain areas originally thought to be “literalist” in their representation of the environment are actually accomplices in the construction of illusory views. While such synthetic activity occasionally leaves us prey to optical illusions, it's a small price to pay for what we get in return: a seamless understanding of often fractured perspectives.

